# Plant Toxin-Based Immunotoxins for Cancer Therapy: A Short Overview

**DOI:** 10.3390/biomedicines4020012

**Published:** 2016-06-01

**Authors:** Letizia Polito, Alice Djemil, Massimo Bortolotti

**Affiliations:** Department of Experimental, Diagnostic and Specialty Medicine-DIMES, Alma Mater Studiorum, University of Bologna, Via San Giacomo 14, 40126 Bologna, Italy; alice.djemil2@unibo.it (A.D.); massimo.bortolotti2@unibo.it (M.B.)

**Keywords:** immunotoxin, immunotherapy, plant toxins, ribosome-inactivating proteins, cancer therapy

## Abstract

Immunotoxins are chimeric proteins obtained by linking a toxin to either an intact antibody or an antibody fragment. Conjugation can be obtained by chemical or genetic engineering, where the latter yields recombinant conjugates. An essential requirement is that the target molecule recognized by the antibody is confined to the cell population to be deleted, or at least that it is not present on stem cells or other cell types essential for the organism’s survival. Hundreds of different studies have demonstrated the potential for applying immunotoxins to many models in pre-clinical studies and in clinical trials. Immunotoxins can be theoretically used to eliminate any unwanted cell responsible for a pathological condition. The best results have been obtained in cancer therapy, especially in hematological malignancies. Among plant toxins, the most frequently employed to generate immunotoxins are ribosome-inactivating proteins, the most common being ricin. This review summarizes the various approaches and results obtained in the last four decades by researchers in the field of plant toxin-based immunotoxins for cancer therapy.

## 1. Introduction

More than one century ago, the Nobel Prize laureate Paul Ehrlich postulated the “magic bullet concept”: drugs that go straight to their intended target cells. Since then, the idea of a drug selectively able to affect only the cells to be eliminated continues to fascinate thousands of researchers around the world.

Inspired by the magic bullet concept, one of the most popular research approaches is based on the linking of a pharmacologically active molecule to a carrier for selective delivery to target cells. Many different molecules have been exploited as carriers and as toxic moieties; the most studied are summarized in Table 1.

The pharmacologically active conjugates are primarily applied to research in the field of cancer therapy [1]. This research arose from the clinical use of radio- and chemotherapeutic agents, which have demonstrable anti-tumor effects but also limitations (considerable side effects due to the lack of selectivity for tumor cells, development of drug resistance, and occurrence of secondary malignancies). As a consequence, the study and development of alternative therapies, such as immunotherapy, were deeply stimulated in order to find therapies with greater specificity for transformed cells and less non-specific toxicity.

Various immunotherapeutic approaches are mainly focused on the identification of specific antigens on the surface of cancer cells. An essential requirement for this approach is that the target molecule is confined to the cell population to be destroyed, or at least that it is not present on stem cells or other cell types essential for the organism’s survival. Antibodies are the most utilized carriers due to their stability in blood and avidity and affinity for their target antigen.

Both bacterial and plant toxins have been used as toxic moieties. The most commonly used bacterial toxic moieties are the *Pseudomonas* exotoxin and the diphtheria toxin, both of which inactivate elongation factor 2 (EF-2), causing irreversible protein synthesis arrest and subsequent cell death. Among plant toxins, ribosome-inactivating proteins (RIPs) are the most common. The use of toxins as pharmacologically active moieties of conjugates has some advantages in comparison with drugs, radionuclides, and enzymes:

1. As opposed to drugs that act in a stoichiometric ratio and only on dividing cells, toxins exert their action in a catalytic manner, do not induce drug resistance, and are able to kill cells in both dividing and quiescent states.
2. Radionuclides have the advantage of eliminating tumor cells that do not express the antigen or express a mutated antigen, but they also have different disadvantages, such as the unspecific toxicity for normal cells that surround the tumor, and the difficulties related to their manipulation, stability, and decay time.
3. In comparison to human enzymes, conjugated toxins have a much greater stability and activity against target cells.

## 2. Immunotoxins (ITs)

Immunotoxins (ITs) are chimeric proteins that are generally obtained by linking a toxin to an intact antibody or a fragment of an antibody. When toxins are linked to other carriers, they are more commonly referred to as “chimeric toxins” or “conjugates”.

IT technology is the culmination of a therapeutic strategy originally devised by Köhler and Milstein in 1975 [2], in which hybridoma technology was introduced, allowing for large-scale production of monoclonal antibodies (mAbs) in mice. Recently, the development of recombinant DNA techniques permitted the generation of chimeric/humanized antibodies and engineered antibody fragments [3,4]. Thanks to numerous and continuous technical advances in the production of new mAbs and related fragments over the last decades, antibody-based immunotherapy has become a fast-growing field in cancer therapy, which has led to important achievements [5,6]. The clinical success of the chimeric (human-murine) anti-CD20 mAb rituximab, the first approved mAb for cancer treatment [7,8], has prompted interest in the development of mAb-based technologies, including ITs.

Until now, hundreds of studies have demonstrated the potential for IT application in many different models, both in pre-clinical studies and in clinical trials [9,10,11,12]. Theoretically, ITs could be used to eliminate any unwanted cell that is responsible for a pathological condition. Most ITs have been prepared to attack cancer cells, endothelial cells of tumor vasculature, immunocompetent cells, or virus-infected cells. The best results are in cancer therapy, especially hematological malignancies. Due to vascular accessibility, hematological cancers have a favorable setting for IT treatment. Furthermore, hematological cells are ideal targets for antibody-based immunotherapy due to the presence of clusters of differentiation (CD) on the cell surface.

The efficiency of an IT in killing cells depends not only on the specific properties of the toxin and the carrier but also on characteristics of the target cell, including antigen density, binding affinity, and intracellular routing. Moreover, immunotherapy specificity is based on characteristics (surface antigens) that are completely independent from those associated with chemotherapy and radiotherapy. This specificity results in fewer side effects for non-target cells and enhanced cytotoxicity toward cell clones resistant to chemotherapy and radiotherapy.

Crucial to the design of an IT is the concerted effort of clinicians (to determine medical needs and models), immunologists (to select the most suitable mAbs), and basic scientists/pharmacologists (to choose the appropriate toxin and linker). An antibody and toxin can be conjugated by means of chemical linkage or by genetic engineering to obtain recombinant conjugates [13]. The choice of the modality to chemically link the antibody and the toxin is another fundamental step in IT design. In fact, the efficacy of an IT mainly depends on its capacity to deliver its toxic moiety into the target cell. The linker must meet some basic requirements: (i) to not impair the antigen-binding capacity of the carrier; (ii) to be stable in the plasma; (iii) to not release the toxin in the extracellular environment; and (iv) to release the toxin intact and in the cellular compartment where it can exert its enzymatic activity. To this end, a disulfide bridge is the most commonly used chemical linkage because such a bond exists in nature between the toxic and lectin subunits of toxins (see below). The presence of thio-disulfide oxidoreductase enzymes in the lysosome allows for breaking the bond and the subsequent release of the toxic payload. A disulfide bridge is obtained upon reaction of the antibody and toxin, previously derivatized using heterobifunctional reagents that are able to introduce thiol reactive groups in the two molecules. The most commonly used heterobifunctional reagents, able to insert thiol groups, are 2-iminothiolane and succinimidyl 3-(2-pyridyldithio)propionate. These reagents bind amino groups accessible on proteins, forming stable amide or imino bonds [14].

Recombinant ITs (also defined as second generation, in contrast with chemically obtained ITs, which are sometimes defined as first generation) are hybrid molecules in which the carrier and the toxin are fused by recombinant DNA techniques to obtain conjugates of smaller size to facilitate penetration into tumor masses [15]. The carrier moiety can be a single-chain variable fragment or a disulfide-stabilized variable fragment, in which a disulfide bond connects the two variable regions. The DNA sequences coding for the toxic agent and the ligand are cloned and expressed in yeast, bacteria, Chinese hamster ovary cells, or insect cells. The ITs produced in bacteria usually accumulate to high levels in “inclusion bodies”, inside which ITs are often incorrectly folded, resulting in less active ITs compared to first generation ITs [16].

The main side effects reported in clinical trials with ITs are immunogenicity and vascular leak syndrome. The generation of anti-toxin antibodies can represent a serious obstacle in the continuation of treatment, mainly in carcinoma and sarcoma patients treated with ITs. Instead, in patients affected by hematological cancers, this side effect is less important, as these patients are often immunosuppressed [13]. Vascular leak syndrome is a consequence of a direct damage toward endothelial cells and is associated with fluid extravasation with edema, hypotension, and in severe form, signs of pulmonary and cardiovascular failure [17].

## 3. Ribosome-Inactivating Proteins

RIPs are a class of toxic enzymes that are widely distributed in the plant kingdom. RIP activity was first identified in rRNA *N*-glycosylase (EC 3.2.2.22) of the rat ribosome; RIPs specifically remove the A4324 adenine residue inside the GAGA sequence on the ricin/sarcin region of the 28S rRNA in the 60S subunit. Importantly, the GAGA sequence is universally conserved among eukaryotic rRNA. Adenine removal damages ribosomes in an irreversible manner, causing inhibition of protein synthesis [18]. RIPs also have *in vitro* *N*-glycosylase activity on other substrates, such as DNA, mRNA, tRNA, and poly(A). For this reason, the definition of RIP enzymatic activity has been proposed to be changed to polynucleotide:adenosine glycosylase [19,20,21].

RIPs are mainly classified as type 1, consisting of a single-chain protein with enzymatic activity, or type 2, consisting of an enzymatic A-chain linked by a disulfide bond to a lectin B-chain that is able to bind to sugar-containing receptors on the cell membrane. The presence of the B-chain in type 2 RIPs allows for rapid internalization of the toxin into the cell. Inside the cell, the two moieties are separated and the active A-chain can enter the cytosol to exert its action. For this reason, most type 2 RIPs are quite toxic. However, a number of non-toxic type 2 RIPs were found in some plant species belonging to the *Sambucus* genus [22].

Despite the different toxicity reported in animals and cell systems [23], both type 1 and type 2 RIPs often show a similar activity on ribosomes in a cell-free system [24].

## 4. Plant Toxin-Based Immunotoxins

Despite a highly efficient cell-killing mechanism, toxins are limited by a lack of specificity. The mAbs have high selectivity for their target, but low cytocidal capacity. Both of these limitations can be overcome by conjugating the two moieties, thus building an IT.

**Figure 1:**
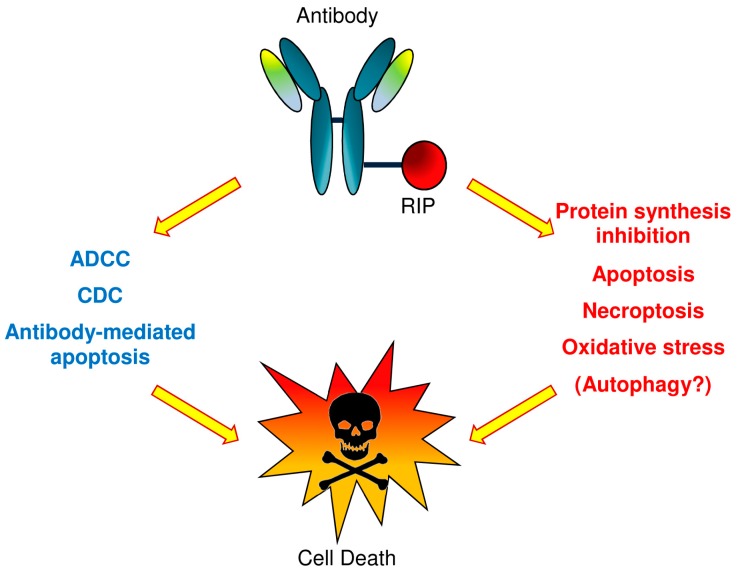
Plant toxin-based immunotoxins (type 1 RIPs or type 2 RIP A chains) can cause cell death by triggering multiple death pathways. Once the toxin reaches the cytosol, endoplasmic reticulum or nucleus, it can cause apoptosis activation, necroptosis, oxidative stress, the inhibition of protein synthesis and probably autophagy. Also, the antibody can activate cell death through apoptosis or through complement-dependent cytotoxicity (CDC) and antibody-dependent cellular cytotoxicity (ADCC), in the event that whole antibody molecules are used.

An IT can trigger cell death by multiple death pathways (see Figure 1). Once the RIP reaches the cytosol, endoplasmic reticulum, or nucleus, it can induce activation of apoptosis, necroptosis, oxidative stress, as well as protein synthesis inhibition and potentially autophagy [25,26]. Moreover, the antibody can also activate cell death through apoptosis, or when whole antibodies are used, through complement-dependent cytotoxicity (CDC) and antibody-dependent cellular cytotoxicity (ADCC).

Ricin is the most commonly used type 2 RIP. The first ITs were prepared with ricin, binding the whole toxin to the mAb, with the hope that antibody avidity and specificity would be able to confer to the conjugate a specific toxicity for the cell target much greater than the non-specific toxicity given by the B-chain. Despite having high efficiency, the high non-specific toxicity of these ITs has prevented their clinical use. Attempts to limit non-specific toxicity involve blocking, modifying, or deleting the lectin chain. Blocking the galactose binding domain in the B-chain was obtained by different means: (a) *in vitro*, by co-administration of excess of lactose or galactose in the culture medium; (b) by reaction with specific ligands, obtained by chemical modification of oligosaccharides; (c) by chemical bond with mAbs that sterically hinder binding to oligosaccharides [27].

A different approach is the use of only the A-chain, obtained by purification after chemical reduction of the A-B disulfide bridge or by genetic engineering. A large number of ITs have been constructed with the ricin A-chain (RTA). However, the use of RTA has also presented some problems, especially related to the difficulty and danger of its purification and non-specific toxicity due to the recognition of residues of mannose present on the A-chain by receptors present on many cell types. To address the problem of non-specificity, different ITs were prepared with the deglycosylated ricin A-chain (dgRTA).

Type 1 RIPs are also commonly used to obtain ITs. In comparison with RTA, type 1 RIPs are more stable, less dangerous, and easier to manipulate. Moreover, a wide variety of well-characterized type 1 RIPs is available, and often, they are immunologically not correlated with each other. These characteristics allow the selection of proteins with different properties that can be very useful in the construction of an IT, such as low systemic toxicity, high stability, *etc*.

The most studied type 1 RIPs for clinical applications as toxic components of ITs are PAP (pokeweed antiviral protein), gelonin, and above all, saporin-S6 [9,11,28]. The success of saporin-S6 derives from its strong activity both in cell-free systems and in cell lines. Moreover, it is extremely resistant to denaturation, high temperature, repeated freeze-thaw cycles, and proteolysis. Saporin-S6 is also stable with respect to chemical modifications, such as those required for derivatization and conjugation [29].

Despite the large number of research studies *in vitro* [30,31,32,33,34,35] or in animal models [11,36,37,38,39], a limited number of clinical trials have been conducted with type 1 RIP–containing ITs in comparison to dgRTA- or RTA-containing ITs (Table 2). Nevertheless, some type 1 RIPs, such as saporin, would have many advantages with respect to ricin, in terms of stability and efficacy, as described above [26]. Altogether, these characteristics render saporin-S6 an attractive molecule for the construction of immunoconjugates.

## 5. Conclusions

Although more than a century has passed from Erlich’s initial idea, the concept of ITs as a “magic bullet” for cancer therapy has not yet faded. In fact, new strategies to study and apply these hybrid molecules are underway, thanks to the availability of new generations of antibodies, such as chimeric or humanized molecules that are already used in clinical therapy. The great interest of institutional researchers and pharmaceutical companies in the field is also demonstrated in the number of patents protecting immunoconjugates or their components. It is the opinion of many investigators that immunoconjugates will likely become important players in cancer treatment in the near future (e.g., [63]). Recently, an anti-IL-2R IT containing dgRTA has been approved by the US Food and Drug Administration for the treatment of cutaneous T-cell lymphoma in adults [13,53]. The large number of antibody–drug conjugates and ITs in clinical trials supports the maturity of this approach [64].

## Figures and Tables

**Table 1:** Components of pharmacologically active conjugates.

Carrier	Toxic Moiety
Antibodies	Drugs
Grow factors	Radioisotopes
Cytokines	Toxins
Hormones	Human enzymes
Antigens	-
Lectins	-

**Table 2:** Clinical trials with plant toxin-containing immunotoxins for cancer therapy ^#^.

Immunotoxin	Antigen Target	RIP	Diseases	Clinical Phase	Ref.
VB6-845	EpCAM	deBouganin	Carcinoma	I	[40]
HuM-195/rGel	CD33	r-Gelonin	Leukemia	I	[41]
B43-PAP	CD19	PAP	ALL	I/II	[42]
H65-RTA	CD5	RTA	CTCL	I	[43]
T101-RTA	CD5	RTA	CLL	I	[44]
XOMAZYME-MEL	HMW mel. ag	RTA	Melanoma	I	[45,46]
Combotox	CD19/CD22	dgRTA	ALL, NHL	I	[47,48]
3A1-dgRTA	CD7	dgRTA	T-LGL, ALL	II	[49]
IgG-HD37-dgA	CD19	dgRTA	NHL	I	[50]
Xomazyme-791	72 kDa gp	dgRTA	Colon cancer	I	[51,52]
RFT-5-dgA	IL-2R (CD25)	dgRTA	CTCL, Melanoma	I/II	[53]
Anti-B4-bR	CD19	bRicin	NHL, Multiple myeloma	I/II	[54,55]
N901-bR	CD56	bRicin	SCLC	I	[56]
260F9-rA	55 kDa gp	RTA	Breast cancer	I	[57,58]
Ber-H2-SO6	CD30	Saporin-S6	HD	I/II	[59,60]
F(ab’)2 BsAb	CD22	Saporin-S6	NHL	I	[61,62]
4KB128 + HD6	CD22	Saporin-S6	BCL	I	[63]

^#^ For more details about clinical trials, see the specific references. ALL: acute lymphoblastic leukemia; BCL: B-cell lymphoma; CD: cluster of differentiation; CLL: chronic lymphoblastic leukemia; CTCL: cutaneous T-cell lymphoma; dgRTA: deglycosylated ricin A chain; gp: glycoprotein; HD: Hodgkin’s disease; NHL: non-Hodgkin’s lymphoma; PAP: pokeweed antiviral protein; RTA: ricin A chain; RIP: ribosome-inactivating protein; SCLC: small cell lung carcinoma; T-LGL: T-cell large granular lymphocyte leukemia.

## Abbreviations

The following abbreviations are used in this manuscript:

ALL: acute lymphoblastic leukemia; BCL: B-cell lymphoma; CD: cluster of differentiation; CLL: chronic lymphoblastic leukemia; CTCL: cutaneous T-cell lymphoma; dgRTA: deglycosylated ricin A chain; gp: glycoprotein; HD: Hodgkin’s disease; IT: immunotoxin; mAb: monoclonal antibody; NHL: non-Hodgkin’s lymphoma; PAP: pokeweed antiviral protein; RTA: ricin A chain; RIP: ribosome-inactivating protein; SCLC: small cell lung carcinoma; T-LGL: T-cell large granular lymphocyte leukemia.
